# Symptoms are known by their companies: towards association guided disease diagnosis assistant

**DOI:** 10.1186/s12859-022-05032-y

**Published:** 2022-12-22

**Authors:** Abhisek Tiwari, Tulika Saha, Sriparna Saha, Pushpak Bhattacharyya, Shemim Begum, Minakshi Dhar, Sarbajeet Tiwari

**Affiliations:** 1 Department of Computer Science and Engineering, Indian Institute of Technology, Patna, Patna, India; 2 Department of Computer Science, University of Liverpool, Liverpool, England United Kingdom; 3 Department of Computer Science and Engineering, Indian Institute of Technology, Bombay, Mumbai, India; 4 Department of Computer Science and Engineering, Government College of Engineering and Textile Technology, Berhampore, Berhampore, India; 5 Department of Medicine, All India Institute of Medical Sciences, Rishikesh, Rishikesh, India; 6 Department of Medicine, Midnapore Homoeopathic Medical College and Hospital, Midnapore, India

**Keywords:** Symptom investigation, Symptom association, Disease diagnosis assistant, Deep reinforcement learning, Early diagnosis, Task oriented dialogue system

## Abstract

Over the last few years, dozens of healthcare surveys have shown a shortage of doctors and an alarming doctor-population ratio. With the motivation of assisting doctors and utilizing their time efficiently, automatic disease diagnosis using artificial intelligence is experiencing an ever-growing demand and popularity. Humans are known by the company they keep; similarly, symptoms also exhibit the association property, i.e., one symptom may strongly suggest another symptom’s existence/non-existence, and their association provides crucial information about the suffering condition. The work investigates the role of symptom association in symptom investigation and disease diagnosis process. We propose and build a virtual assistant called Association guided Symptom Investigation and Diagnosis Assistant (A-SIDA) using hierarchical reinforcement learning. The proposed A-SIDDA converses with patients and extracts signs and symptoms as per patients’ chief complaints and ongoing dialogue context. We infused association-based recommendations and critic into the assistant, which reinforces the assistant for conducting context-aware, symptom-association guided symptom investigation. Following the symptom investigation, the assistant diagnoses a disease based on the extracted signs and symptoms. The assistant then diagnoses a disease based on the extracted signs and symptoms. In addition to diagnosis accuracy, the relevance of inspected symptoms is critical to the usefulness of a diagnosis framework. We also propose a novel evaluation metric called Investigation Relevance Score (IReS), which measures the relevance of symptoms inspected during symptom investigation. The obtained improvements (Diagnosis success rate-5.36%, Dialogue length-1.16, Match rate-2.19%, Disease classifier-6.36%, IReS-0.3501, and Human score-0.66) over state-of-the-art methods firmly establish the crucial role of symptom association that gets uncovered by the virtual agent. Furthermore, we found that the association guided symptom investigation greatly increases human satisfaction, owing to its seamless topic (symptom) transition.

## Introduction

**Fig. 1:**
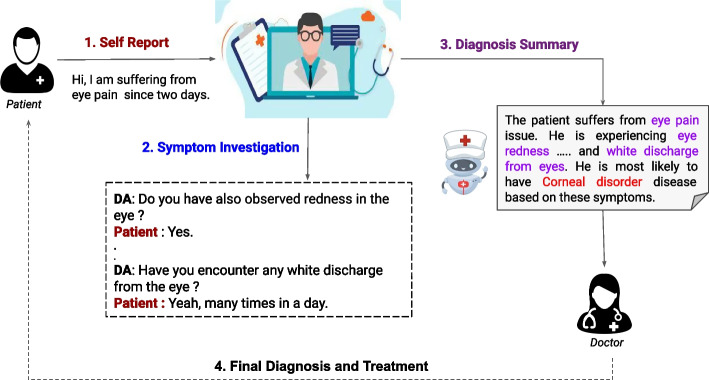
An illustration of an automatic disease diagnosis system —There are three key stages, namely self reporting by a patient, symptom investigation through conversation, and disease inference depending on extracted symptoms

Diagnosis is the primary and crucial stage of any medical treatment process, during which doctors investigate, analyze symptoms, and identify patients’ diseases. As reported by the World health organization (WHO), 2013 [1], the world falls short of 7.2 millions medical workers, which is expected to reach 12.9 millions in the upcoming decade. The dearth still continues as per a report by WHO, 2019 [2], there are many countries where doctor per 1000 people is less than one. These figures firmly suggest the betterment of the healthcare system by increasing health workers and utilizing their time more efficiently and critically. As a result, there is a surge of interest in utilizing Artificial Intelligence (AI) based systems to reduce the workload of medical professionals [3]. One of such manifestations is automatic diagnosis with the help of a virtual agent that can conduct a thorough symptom investigation and present a detailed report to doctors. Some automatic disease diagnosis systems such as Mayo Clinic 1, Babylon Healthcare 2 and GMAN are already deployed, which are being extensively used by both hospitals and end-users. A study conducted by Fox et al. [4] showed 35% U.S adults had utilized self-diagnosing tools before consulting with real doctors. A typical diagnosis process has been illustrated in Fig. 1.

Diagnosis system deals with exponential state space [5], and the diagnosis assistant is expected to learn a mapping of these states to appropriate diseases. Consider a diagnosis system having *n* number of symptoms and *D* number of diseases. A patient may have any combination of these *n* symptoms, i.e., a subset of these symptoms. With *n* number of elements in a set, there can be 2$^{n}$-1 sub-sets (excluding null set). Thus, the diagnosis assistant learns to map symptom space of size 2$^{n}$-1 to disease space (D). Also, there are many overlapping symptoms across diseases, which increases the problem complexity by many folds [6]. Thus, an intelligent and robust symptom investigation by virtual agents is key for diagnosing patients correctly and efficiently. In real world, a considerably large number of diseases are diagnosed by doctors through only an in-depth symptom investigation [7]. In some cases, they need further evidence through laboratory reports to reach a conclusive diagnosis. Nevertheless, symptom investigation is essential and crucial for suggesting an appropriate lab examination.

In recent few years, significant efforts have been made to build an adequate and robust disease diagnosis assistant [8]. Such virtual assistants’ primary responsibility is to aid doctors and conduct symptom investigations [9]. When we consult our health issues with doctors, they do not usually infer a condition/disease based on only our informed symptoms and signs. They investigated further symptoms and signs to reach a conclusive disease. In [10], authors have developed a task-oriented dialogue system [11] that extracts signs/symptoms in addition to patient self-report through conversation. Most of the existing disease diagnosis assistants [12, 13] are built upon this fundamental work. Some of them focus on technique improvement, such as the incorporation of hierarchical reinforcement learning (HRL) [12] and generative adversarial network (GAN) [14] while others aim to investigate some fundamental research questions [5]. Since diseases are described by a set of symptoms, an understanding of the association between these symptoms can significantly influence both symptom investigation and disease identification. Furthermore, the association guided symptom inspection can significantly enhance user satisfaction because of the seamless topic (symptom) transition. However, none of the existing diagnostic works [10, 12, 15] have investigated the role of symptom association and leveraged the information in disease diagnosis. Motivated by the research gap, we aim to investigate the efficacy of symptom association in disease diagnosis and build a symptom association-guided disease diagnosis assistant.

It is well said that a man is known by the company he keeps [16]. It has also been observed to be true for words [17], which later became key for developing different word embedding techniques such as Word2Vec [18]. In addition to the presence of suffering symptoms (s$_i$, s$_j$) in the set of observed symptoms (OS), the co-occurrence of these symptoms (s$_i$, s$_j$ $\in$ OS) provides vital and distinguishable information for determining the patients’ disease. Here, *OS* is the set of observed symptoms. The appropriateness and relevance of inspecting symptoms directly affect patients’ experience with the system. Thus, a symptom association guided investigation and diagnosis can improve both diagnosis efficacy and patients’ experience. To the best of our knowledge, this work is the first attempt to investigate the role of symptom association and model the key information in the learning process of a virtual agent responsible for automated disease diagnosis. The proposed virtual agent conducts a symptom association-guided symptom investigation and extracts symptoms and signs through a conversation with patients. Once symptom investigation completes, it diagnoses patients based on the status of investigated symptoms.

The primary objectives of any autonomous disease diagnosis system are to diagnose patients accurately and efficiently [19]. The end-users experience with it also determines its effectiveness and usability. Thus, in addition to the final outcome (diagnosis accuracy), the diagnosis assistant’s behavior with patients is also a paramount concern. A single irrelevant symptom request can substantially impact patients’ trust in the system. For instance, two automatic diagnosis systems, S$_1$ and S$_2$, both diagnose a patient’s disease *D* accurately (in equal time); system S$_1$ will be preferred if system S$_1$’s relevance of symptom investigation is higher than the other. However, the existing systems [10, 12, 15, 19] have overlooked this key aspect that determines system’s efficacy and usability in real-world setting. We propose a new evaluation metric called *Investigation Relevance Score (IReS)* which measures the relevance of conducted symptom investigations in relation to patient’s chief complaints and conversation contexts. The main contributions of this work are as follows:

- The work investigates the role of symptom association in diagnosis process and proposes a novel association-guided symptom investigation incorporated virtual assistant responsible for automated diagnosis using hierarchical reinforcement learning.
- We propose a new evaluation metric called Investigation Relevance Score (IReS), which measures the relevance of symptom investigation conducted by an automatic diagnosis system.
- The proposed model outperforms several baselines and state-of-the-art diagnosis assistants in all evaluation metrics, including human evaluation scores, and achieves state-of-the-art performance.

The paper is organized as follows: The related work section describes existing relevant works on disease diagnosis virtual assistants. The methodology section explains and illustrates the proposed symptom association guided disease diagnosis model. We describe the utilized synthetic diagnosis dataset (SD) in the dataset section. The experimental setup and parameter values are provided in the experimental setup section. We report and discuss the obtained results in the result and discussion section. In the case study and analysis section, we discuss some case studies of the performances of different diagnosis assistants. We conclude by summarizing the work and outlining some potential directions for future work.

## Related work

The existing works on automatic disease diagnosis can broadly be categorized into two groups: 1. Disease prediction systems [20], which aim to predict a disease for a given patient’s medical data, such as X-ray report. 2. Automatic disease diagnostic systems [12], which conduct a symptom investigation and diagnose patient’s disease depending upon the status of investigated symptoms. The proposed work belongs to the second category. The work is mainly related to the following three research areas: Electronic health records, Automatic disease diagnosis systems, and Automatic disease diagnosis dialogue systems. We have summarized the relevant works and their limitations in the subsequent paragraphs.

### Electronic health records

In the early 2000s, Electronic health records (EHR) [21, 22] based systems were proposed with the motivation of assisting patients in rural areas by virtual means. However, an EHR system requires multiple devices and their synchronization [23]. To overcome such dependencies and intensive efforts, researchers have introduced a new paradigm for automatic disease diagnosis (for non-fatal/sensitive diseases), where an interactive system conducts a thorough symptom investigation and diagnoses patients’ disease based on extracted symptoms [24]. The work [25] describes the development of a deep learning model called DDxNet for diagnosing diseases from time-varying clinical data having different modalities such as ECG, EEG, and EHR. Chakraborty et al. [26] proposed an ensemble feature selection that combines multiple machine learning classifiers such as K-Nearest Neighbors Bagging Technique (KNNBT) and Neural Network Bagging Technique (NNBT) for selecting an effective set of features from bio-medical datasets. In [27], the authors have proposed a deep learning-based smart healthcare system for heart disease prediction. The model utilizes both sensor and EHR data for patient context representation, which achieves state-of-the-art performance for the diagnosis task.

### Automatic disease diagnosis systems

Tang et al. [24] have proposed an ensemble neural network model for symptom checking and diagnosis, which consists of many small models for different anatomical parts, leading to superior performance compared to existing traditional monolithic systems. However, the system utilizes a rule-based module for selecting different anatomical networks, making it harder to be adapted to another diagnosis system. Peng et al. [15] incorporated a novel feature rebuilding technique in the diagnosis process, which directly includes implied symptoms rather than enquiring explicitly. This feature rebuilding technique needs huge manual labor and analysis for an extensive diagnostic system with many common symptoms across multiple diseases; otherwise, a trivial rebuilding technique may degrade performance. In real life, doctors’ investigation also depends on patients’ personal information, such as age and gender. Motivated by such scenarios, Kao et al. [13] have proposed a context-aware symptom checker, which showed that context (patient’s personal information) such as patient’s gender and age provide key guidance in conducting an appropriate and efficient diagnosis. In [28], the authors have proposed a machine learning-based model, which identifies the possibility of both diabetes and liver disease from patient data. Autonomous heart disease prediction is one of the most focused concern of bio-medical research community. Chakraborty et al. [29] introduced a fog-based heart disease prediction model, which significantly improved both diagnosis time and accuracy. In [30], the authors have proposed an ensemble-based machine learning model which predicts several fatal diseases, including hepatitis and liver disorder.

### Automatic disease diagnosis dialogue systems

In real world, doctors diagnose a considerably large number of diseases through only an in-depth symptom investigation. Motivated by the real-world scenario, Wei et al. [10] formulated diagnosis as a task-oriented dialogue system problem, which illustrated and emphasized the role of implicit symptoms extracted by the dialogue agent in addition to patient-reported symptoms for accurate diagnosis. Doctors’ prior learning is crucial for their appropriate behavior for both diagnosis and treatment. To infuse such intelligence, Xu et al. [31] have proposed a knowledge routed relational dialogue system (KR-DS) that utilizes a rich medical knowledge graph (disease-symptom) in the learning process. Liao et al. [12] have introduced an integrated and synchronized two-level policy framework using hierarchical reinforcement learning [32], which outperformed the flat policy approach [10] by a significant margin, demonstrating the efficacy of disease group aware symptom investigation. Liu et al. [33], developed a conversational medical corpus having conversations between clinicians and users. They also proposed a novel medical entity controlled medical response generation model that performs superior to existing non medical entity controlled generation models. The work [34] presents a low cost millimeter antenna for building portable 5G communication gadgets. In [9], the authors propose a variant of deep Q network (DQN) called prototype deep Q network that quickly adapts to new or rare diseases having a handful number of samples.

In real life, doctors also learn from external knowledge, such as symptom-disease relational databases. Motivated by the observation, the work [35] proposed a context-aware knowledge-infused virtual assistant that generates relevant and context-aligned responses. In real life, doctors continuously exploits extracted symptom information for intelligent symptom inspection. They hypothesize a set of probable diseases based on extracted symptoms and first inspects the potential symptoms of these candidate diseases. Tiwari et al. [5] investigated the idea and proposed a knowledge-infused context-driven (KI-CD) disease diagnosis model that inherits the doctors’ diagnosis behavior. The obtained performance by the KI-CD model firmly illustrates the effectiveness of the principle and accomplishes state-of-the-art performance. In many cases, we find it difficult to describe some of our signs and symptoms, such as mouth ulcers, through text. Thus, we often leverage visual means to describe them. Inspired by the effectiveness of visual modality in symptom investigation and diagnosis, the work [36] proposed a multimodal disease diagnosis assistant that extracts symptoms from both textual and visual responses of end-users. The study found that incorporating visual modality into symptom investigation and disease diagnosis enhanced both diagnosis accuracy and end-user satisfaction significantly.

## Methodology

**Fig. 2:**
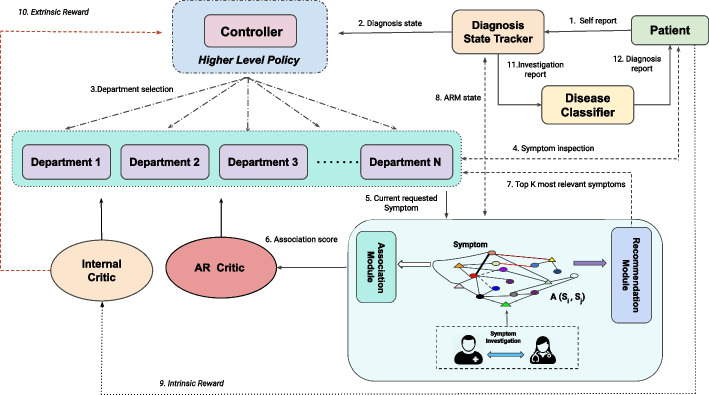
Proposed *Association guided Symptom Investigation and Diagnosis Dialogue System* (A-SIDDS) where the controller and department policies conduct symptom investigation guided by symptom-symptom association /recommendation and disease classifier diagnoses patient as per the symptom investigation report

In a typical diagnosis, clinicians undertake a symptom investigation and diagnose a disease based on observed symptoms (Fig. 1). The detailed architecture of the proposed dialogue system, A-SIDDS (association guided symptom investigation and diagnosis dialogue system), is illustrated in Fig. 2. A patient initiates the diagnosis process by informing their suffering symptoms (explicit symptoms). The controller policy of the proposed dialogue system acts as a clinic receptionist, which activates a lower-level department policy as per the patient report. The activated departmental policy conducts a symptom investigation guided by Association and Recommendation Module (ARM). Once the lower-level policies collect adequate information, the controller policy activates the disease classifier, which diagnoses patients’ diseases depending on the collected information. The detailed working methodologies of each module are as follows:

### Symptom investigation

Symptom investigation is the first stage of diagnosis, where doctors conduct an investigation and extract other relevant symptoms depending upon patients’ reported chief complaints and other confirmed symptoms during inspections. Thus, the agent aims to learn appropriate and intelligent behavior for collecting adequate symptom information in minimal time, i.e., an optimal diagnosis dialogue policy. The policy learning loop consists of three main components: I. Diagnosis Policy Learning, II. Association & relevance module (ARM), and III. Internal & Association and Recommendation (AR) critic. Each sub-stage and its detailed working method are explained below.

### Diagnosis policy learning

Diagnosis policy ($\pi$) is the decision function, which decides whether to investigate symptoms or predict disease after observing symptoms, i.e., $a = \pi (S)$, where *S* is a set of observed symptoms, and *a* could be a symptom or disease. To improve investigation efficacy and patient satisfaction, clinics used to have different departments such as ENT (Ear, Nose, and Throat) and pediatrics, etc. Motivated by the real-world scenario and the promising results obtained by Liao et al. [12, 37], we also utilized a hierarchical policy learning method, where the higher-level policy (controller) activates one of the lower-level policies (departmental) depending on patients’ self-report and other symptoms and the department policy conducts group-specific symptom investigation.

#### Controller policy

Controller policy is the first layer policy, which is responsible for activating an appropriate department policy (DP$_i$) and disease classifier for symptom inspection and disease projection, respectively. It can be seen as a clinic’s receptionist who refers patients to a particular department as per their chief complaint/self-report. It is also responsible for triggering the disease classifier (DC) once the lower policies (department policies) collect adequate symptom information. The controller policy selects an action (*ac*) depending upon current dialogue state (S) as follows: $ac = P (A_c \vert S, \pi _c)$ where $\pi _c$ is the controller policy, $A_c$ is its action space which consists of department policies ($DP_{i}$) and disease classifier. For each action *ac* on a state S, the agent gets a penalty/reward (r$_c$: Reward(S, *ac*)) depending upon the effectiveness of the taken action as follows:

1 $$\begin{aligned} \begin{aligned} rc_{t} = {\left\{ \begin{array}{ll} \sum _{i=1}^{n} \gamma ^{i}_{c} r_{t+i}^{d}, &{} \quad \text {if } ac_t = DP_{i} \\ r_{t}^{d}, &{} \quad \text {if } ac_t = DC \\ \end{array}\right. } \end{aligned} \end{aligned}$$

where *i* is the number of turns taken by the activated lower level policy corresponding to the master action, ac$_t$. The agent aims to maximize the cumulative reward over episodes ($R = \sum _{n=1} ^{N} \sum _{t=0} ^{T} \gamma _{c}^{t} * rc_{t}$), leading to adequate symptom investigation and thus accurate diagnosis. Here, *N*, *T* are the number of dialogues in an episode and the number of turns in n$^{th}$ conversation, $\gamma _c$ is discounted factor which governs the role of immediate and future rewards in policy learning.

The controller policy $\pi _{c}$ is optimized using a value-based deep reinforcement learning technique called Deep Q Network (DQN) [38]. It learns a state-action value function (Q$^c$ (S, ac)), which estimates a value for each action (department) for a given dialogue state S (informed symptoms). The policy selects an action with highest Q value (reward), i.e., $ac = argmax_i {Q^{c} (S, A_{c}^{i} | \pi _c})$. The $Q^c$ function has been calculated and optimized through Bellman equation [39] and temporal difference (TD) loss [40] as follows :

2 $$\begin{aligned} Q^c(S_t, ac_t)= & {} {\mathbb {E}} [rc_t + \gamma _{c} * \text {max}_{ac_{t+1}} Q^c(S_{t+1}, ac_{t+1})] \end{aligned}$$

3 $$\begin{aligned} L_{t}^{c}= & {} [(rc_{t} + \gamma _c * \text {max}_{ac_{t+1} \in A_c} Q^c (S_{t+1},ac_{t+1} \vert \pi _c^{t-1}, \theta ^{t-1})) - Q^{c}(S, a \vert \pi _c^t, \theta _{t} ]^2 \end{aligned}$$

where $L_{t}^{c}$ is the loss at $t^{th}$ time step, which is difference between state-action value calculated through current policy parameter (behavior network : $\theta _t$) and previously froze policy parameter (target network : $\theta _{t-1}$).

#### Departmental policy

The departmental/lower lever policies (DP$_i$: $\pi ^{i}$) are responsible for symptom inspection corresponding to their departments. The proposed model has nine departmental policies corresponding to each disease group. These departmental policies learn to select an appropriate action (symptom for inspection) depending upon the current dialogue state, which contains informed/confirmed symptoms. It selects an action (a$_i$) as follows:

4 $$\begin{aligned} a_i = \text {argmax}_j Q^i (A_{ij} \vert S, \pi ^i) \end{aligned}$$

where $Q^i$ is state-action value function of $i^{th}$ department policy ($\pi ^{i}$) and A$_{ij}$ is j$^{th}$ action of i$^{th}$ departmental policy. The state, *S*, consists of the status of informed and inspected symptoms, dialogue turn, agent’s previous actions, *K* most relevant symptoms predicted by the ARM module, and reward. The size of the action space of each policy is N$_i$ + 1, where N$_i$ is the number of symptoms in i$^{th}$ department. The additional action is to return the control to the controller policy. The department agent gets a reward /penalty (internal and ARM critic) at each time step depending upon the appropriateness and relevance of agent’s action (a$_i$) to the current state (S). These policies ($\pi ^{i}_d$) have also been optimized using the DQN algorithm as the controller policy (Equs. 2 and 3).

### Association and relevance module (ARM)

The Association and Relevance Module (ARM) is responsible for conducting knowledge-aware, association-guided symptom investigation for adequate symptom information extraction. The module gets the current state (S$_t$) and inspected symptom (Sym$_t$) as inputs, and it outputs an association score & symptom recommendation (RS$_t$). The association module provides an association score (as$_t$) depending upon the relevance of the currently requested symptom (Sym$_t$) with the confirmed symptoms (SS), i.e.,

5 $$\begin{aligned} as_t = \sum _{k=1}^{n_t} Association (Sym_t, SS_k) \end{aligned}$$

where *SS* is the set of inspected and confirmed symptoms (including patient self-report) till t$^{th}$ turn of the dialogue and n$_t$ is the number of symptoms in it. The association score is provided as a critic to the agent, reinforcing the agent to conduct an association-aware symptom investigation. We construct and utilize a symptom-symptom knowledge graph to calculate the associations between two symptoms. In the knowledge graph, nodes represent symptoms, and an edge between two nodes signifies the co-relation between these two symptoms. The edge between two nodes/symptoms (S$_i$, S$_j$) is determined based on the frequency of their co-occurrence. The weight of the edge from the symptom $S_i$ to $S_j$ is computed as follows:

6 $$\begin{aligned} Association(S_i, S_j) = \frac{n(S_i, S_j)}{\sum _k n(S_i, S_k)} \end{aligned}$$

where $n(S_i, S_j)$ is the number of instances in the diagnosis dataset, where S$_i$ and S$_j$ have co-occurred. The term *k* ranges in the entire symptom space (Sy). Here, the denominator represents the number of instances where the symptom $S_i$ has occurred with symptom $S_k$ ($S_k$ $\in$ Sy). Thus, the association score of the symptom $S_i$ with $S_j$ signifies the chances of occurrence of $S_j$ with it. The high value of the association score ($S_i$, $S_j$) indicates that a patient is most likely to suffer from symptom $S_j$ if he/she observes symptom $S_i$.

A symptom may strongly suggest the existence of another symptom, which are caused by a common condition. For instance, when we think about cold, the next symptom that comes to our mind is cough. Cold and cough often co-occur together. Motivated by the observation, the proposed model incorporates a recommendation module, which recommends some of the most relevant symptoms (RS) from the entire symptom set (Sy) depending upon confirmed symptoms, *SS*. It selects top *K* symptoms from symptom space, which are highly relevant to the current context (confirmed symptom set, *SS*) and co-occur together. This module utilizes association scores for determining top *K* relevant symptoms as follows:

7 $$\begin{aligned} RS = \Pi _{i=1}^{K} argmax _{s \in Sy} \sum _{j=1}^{|SS|} Association(SS_j, s) \end{aligned}$$

These recommended symptoms are reflected in the current dialogue state, and the agent is reinforced to investigate these most relevant symptoms through the recommendation critic. This module aids the agent in conducting a knowledge-aware, association guided symptom investigation, which improves the user experience and reduces the number of turns required to diagnose the patient.

### Internal and association and recommendation (AR) critics

A reinforcement learning agent’s reward model is one of the most critical elements, which implicitly supervises the agent for the underlying task. We propose and incorporate two novel reward functions, namely recommendation-based critic and association-based critic, to reinforce the agent for conducting context-aware, association-guided symptom investigation. The critics (intrinsic critic: r$_d$, r$_{rr}$: recommendation based critic, and r$_{ar}$: association based critic) are defined as follows:

8 $$\begin{aligned} r_{d}= & {} {\left\{ \begin{array}{ll} = + t_1 * N &{}\text {if success}\\ = + t_2 * N , &{} \text {if } match (Sym_t) = 1 \\ = -t_3 * N, &{} \text {if repetition} \\ = 0, &{} \text {Otherwise} \end{array}\right. } \end{aligned}$$

9 $$\begin{aligned} r_{rr}= & {} {\left\{ \begin{array}{ll} = + t_4 &{}\text {if } Sym_t \in RS_t\\ = - t_5, &{} \text {Otherwise } \end{array}\right. } \end{aligned}$$

10 $$\begin{aligned} r_{ar}= & {} {\left\{ \begin{array}{ll} = + t_4 &{}\text {if } as_t >h\\ = +1 &{} \text {if } l< as_t < h \\ = - t_5, &{} \text {Otherwise} \end{array}\right. } \end{aligned}$$

11 $$\begin{aligned} {{\mathrm {r}}}= &{} {{\mathrm {r}}}_{{\mathrm {d}}} + ({{\mathrm {r}}}_{{\mathrm {rr}}} + {{\mathrm {r}}}_{{\mathrm {ar}}}) \end{aligned}$$

where *N* and $t_i$ are the maximum no. of allowed turns for diagnosis and shaping parameters, respectively. The term, *match*(Sym$_t$) = 1 indicates that the department policy has requested a symptom (Sym$_t$) that the patient is truly suffering from. Here, $Sym_t, RS_t$, and $as_t$ are the agent’s requested symptom, recommended symptoms, and association score between $Sym_t$ and other conformed symptoms (SS) at t$^{th}$ turn, respectively. The terms *l*, *h* denote the lower and desired thresholds for association scores between requested symptom and confirmed symptoms (SS), respectively.

The internal critic (r$_d$) reinforces to complete the task successfully, whereas the immediate rewards (recommendation: r$_{rr}$ and association: r$_{ar}$) act as the task behavior shaping elements. The recommendation and association reward models provide a reward/penalty depending upon the appropriateness of agent action and its relevance in relation to dialogue context (already informed symptoms including patient self-report, SS). If the agent inspects a recommended symptom, it gets a reward (case 9.1); otherwise, it gets a small penalty. The association reward (r$_{ar}$ provides a reward/penalty proportional to the relevance (association score) of the currently requested symptom with the ongoing context/confirmed symptoms (SS), which motivates the agent to enquire relevant and knowledge-grounded symptoms.

### Diagnosis state tracker, patient and disease classifier

Diagnosis state tracker is responsible for tracking dialogue (diagnosis) state, which contains information about inspected symptoms, dialogue turn, and the agent’s previous actions. After each agent and user turn, the state tracker updates dialogue state with essential information such as agent requested symptoms, user response, turn number, and the reward/critic corresponding to agent action. We have developed a pseudo environment/user simulator similar to the popular task-oriented user simulators [12, 41]. The user simulator initializes each diagnosis session with a diagnosis case from training samples. At the first turn of a conversation, the patient simulator informs the diagnosis agent’s self-report (all explicit symptoms) and asks to identify the disease/condition that the patient may be experiencing. Then, the simulator responds to each agent’s request for symptoms as per the sampled diagnosis case during the conversation. Disease classification is the final stage, which diagnoses a disease depending upon the extracted symptoms (including the patient’s self-report). In our work, it is a two-layered deep neural network, which takes a one-hot encoding representation of symptom status as input and predicts the probability distribution over all diseases.

### Investigation relevance score (IReS)

Automatic disease diagnosis is a sequential decision problem in which an agent interacts with end-users over time for symptom investigation and then diagnoses the most appropriate disease based on the observed symptoms. Thus, an adequate set of symptom collection is critical to accurate diagnosis, which directly influences end-users engagement with the system. A single irrelevant symptom inspection by a diagnosis agent may cause end-users to lose trust in the system, resulting in the termination of dialogues in a large number of such cases. For instance, a person comes with difficulty of breathing, and if an agent inspects some irrelevant (less relevant) symptoms such as skin growth and knee swelling, the end-user may become annoyed and terminate the chat. However, the existing works [10, 12, 15, 24] employ objective metrics such as diagnosis accuracy and symptom investigation time for measuring their proposed models’ efficacy. Motivated by the vital significance of symptom relevance and the inability of existing evaluation metrics to capture this critical aspect, we formulate and propose a novel automatic evaluation metric called *Investigation Relevance Score (IReS)* for evaluating a diagnosis agent’s efficacy in terms of the relevance of symptoms inspected by the agent during symptom investigations. The metric is formulated as follows:

12 $$\begin{aligned} I \, ReS-1= & {} \frac{\sum _{i=1}^{m} \sum _{j=1}^{j=n} Association(S_j, PSR_i)}{t* \sum n_i} \end{aligned}$$

13 $$\begin{aligned} I \, ReS-2= & {} \frac{\sum _{i=1}^{m} \sum _{j=1}^{j=n} Association(S_j, SS_{ij})}{t* \sum n_i} \end{aligned}$$

where *m* is the number of testing samples, and $n_i$ is the number of turns taken by the agent for i$^{th}$ diagnosis test sample. The term PSR$_i$ and SS$_{ij}$ denote patient self-report and confirmed symptoms till j$^{th}$ turn of i$^{th}$ sample, respectively. The IReS-1 measures the relevance of symptom investigation with patient self-reported symptoms (PSR), and the IReS-2 measures the relevance of symptom investigation with the ongoing context (confirmed symptoms, including PSR)

## Dataset

**Fig. 3:**
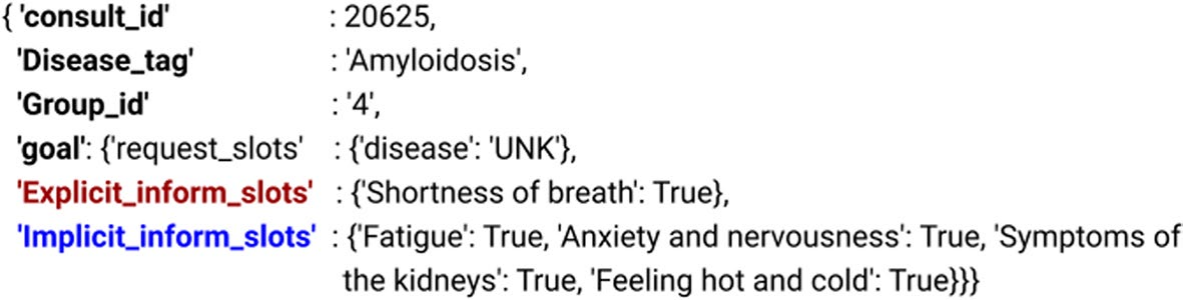
A diagnosis data sample from SD dataset

**Fig. 4:**
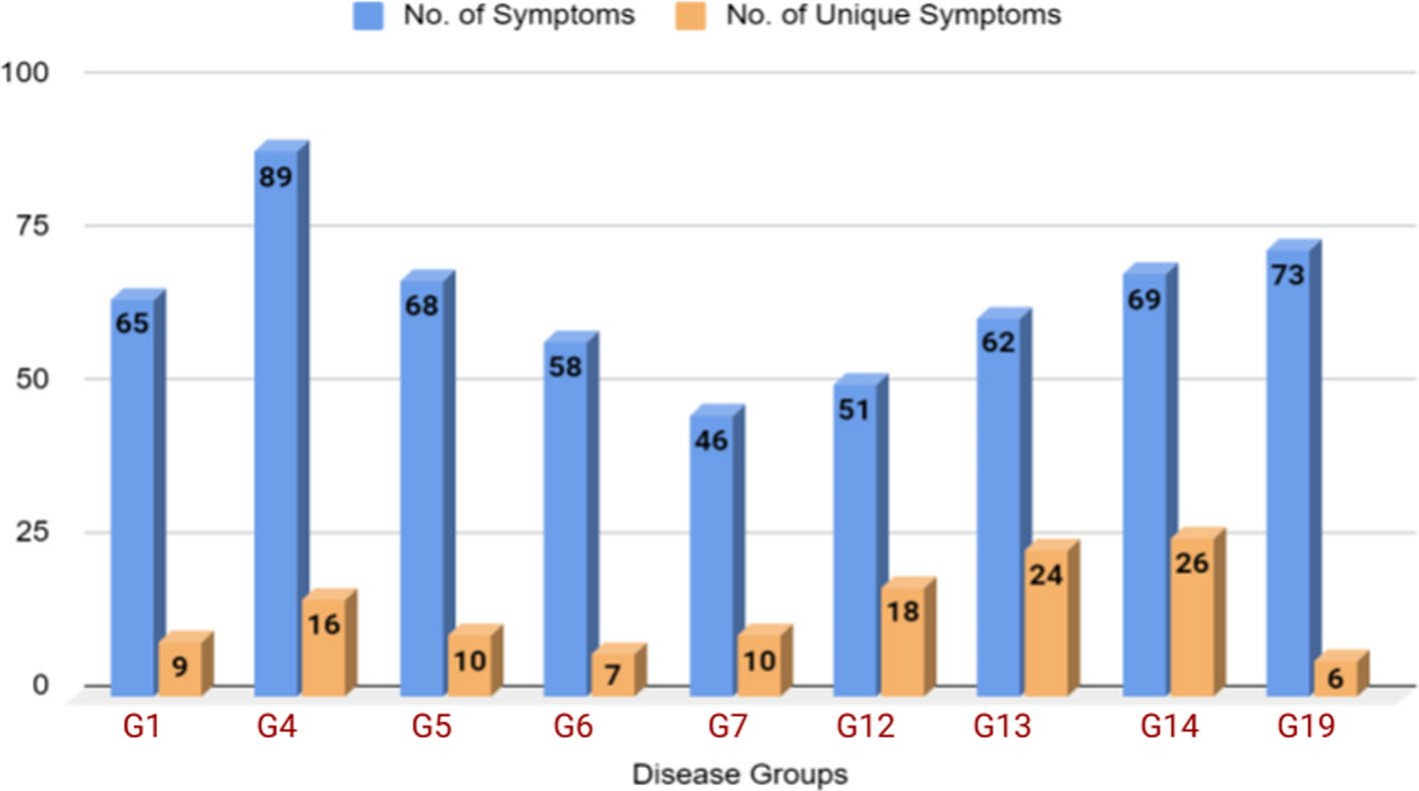
Distribution of the total number of symptoms (blue) and number of unique symptoms (orange) across different groups

We have experimented with the largest publicly available English medical dataset, Synthetic dataset (SD) [12], which contains 30,000 patient samples. A sample diagnosis data and the dataset statistics are reported in Fig. 3 and Table 1, respectively. The distributions of the total number of symptoms & unique symptoms across different groups^3^ are illustrated in Fig. 4.

**Table 1:** SD dataset statistics

Entries	Values
# of diseases	90
# of disease groups	9
# of diseases in each group	10
# of symptoms	266
Average no. of symptoms in self-report	1
Average no. of implicit symptoms	2.6

## Experimental setup

The proposed methodology 4 is trained^5^ and evaluated on 80% (24,000) and 20% (6,000) patients’ samples of the benchmarked dataset, respectively. For sufficient exploration, we have utilized the most popular and well-accepted exploration strategy called $\epsilon$-greedy [42]. When the agent explores, it can improve its current knowledge and gain better rewards in the long run. The model is trained for 5000 epochs, each having 100 dialogues. The values of key hyperparameters are: $\gamma _m$ 0.9, $\gamma _w$ 0.95, learning rate ($\alpha$) 0.0005, {t$_1$, t$_2$, t$_3$, t$_4$, t$_4$, t$_5$ } - {3, 2, 2, 15, 2}, epsilon ($\epsilon$) 0.1, max allowed turn (N) 28, episode size 100, and batch size 100. The epsilon value 0.1 signifies that the agent explores 10% times and exploits 90% (1-$\epsilon$) times. All the hyperparameter values are decided empirically.

## Results and discussion

We utilize the most popular automatic diagnosis evaluation metrics (viz., diagnosis success rate, dialogue length, match rate, match rate2, and disease classifier accuracy) [10, 31, 41] for evaluating our proposed agent’s performance, comparing with state of the art methods and other baselines. Match rate is the ratio of no. of true symptoms (extracted through conversation) to the total number of agent’s symptom requests (query), and match rate 2 is the ratio of the number of true symptoms (extracted through conversation) to the total number of symptoms in patient’s implicit symptom set. The average match rate (AMR) and average match rate 2 (AMR2) are averages of match rate and match rate 2 over dialogues, respectively. These metrics are computed as follows:

- **Success rate** = $\frac{ \sum _{i = 1} ^{i = EL} DS_{i}}{EL}$, where EL (Episode length) denotes the number of simulated dialogues in an episode, $DS_{i} = 1$ if the $i^{th}$ dialogue ends successfully, i.e., the agent informs correct disease, otherwise 0.
- **Avg reward** = $\frac{\sum _{i=1}^{i=EL} \sum _{j = 1}^{j=t} r_{ij}}{EL}$, where r$_{ij}$ represents reward received by agent in j$^{th}$ turn of i$^{th}$ dialogue session of an episode. Here *t* represents the number of dialogue turns in j$^{th}$ dialogue session.
- **Avg dialogue turn** = $\frac{ \sum _{i = 1} ^{i = EL} len_{i}}{EL}$, where $len_{i}$ denotes number of dialogue turns taken by the agent in $i^{th}$ dialogue session of an episode.
- **Average match rate (AMR)** = $\frac{\sum _{i=1}^{i=EL} m_i/r_i }{EL}$ x 100, m$_{i}$ indicates total number of agent’s requested symptom, which belongs to the patient’s suffering symptoms. Here, $r_i$ signifies the total number of symptoms requested by the agent during $i^{th}$ conversation.
- **Average match rate 2 (AMR2)** = $\frac{\sum _{i=1}^{i=EL} m_i/t_i }{EL}$ x 100, m$_{i}$ indicates total number of agent’s requested symptoms, which belongs to the patient’s suffering symptoms. The term $t_i$ denotes the total number of true implicit symptoms of the patient in $i^{th}$ conversation.

We have experimented with two reinforcement learning algorithms, namely DQN and Double DQN (DDQN) [38, 43]. In addition to these standard evaluation metrics, we have also evaluated the models in terms of the proposed metric, *Investigation Relevance Score (IReS)*.

To determine the efficacy of the proposed method, we have compared our proposed model with the following baselines and the current state-of-the-art methods. **i. SVM-ex**: SVM model [44] with only explicit symptoms / patient self report. **ii. REFUEL** The REFUEL model [15] incorporated a novel feature rebuilding technique in the diagnosis process, which directly includes implied symptoms rather than enquiring explicitly. **iii. GAMP** The model [14] utilizes a generative adversarial network (GAN) based symptom investigation methodology where the generator selects an action based on state, and the discriminator evaluates the effectiveness of the chosen action. **iv. KR-DS** The KR-DS agent [31] leverages external medical data (knowledge-graph) for learning an optimal behavior for symptom investigation and disease diagnosis. **v. Flat Policy**: An unified policy [10] that conducts both symptom investigation and diagnosis, **vi. HRL**: A hierarchical policy learning method [12] having master and worker as levels, **vii. PR-SIDDA**: This is our proposed model, which utilizes only patients’ self-reports for conducting an association-guided investigation, **viii. A-SIDDA**: Association guided symptom investigation and diagnosis dialogue system (A-SIDDA), which utilizes patients’ self-reports as well as current context (confirmed symptoms) for conducting symptom investigation.

**Fig. 5:**
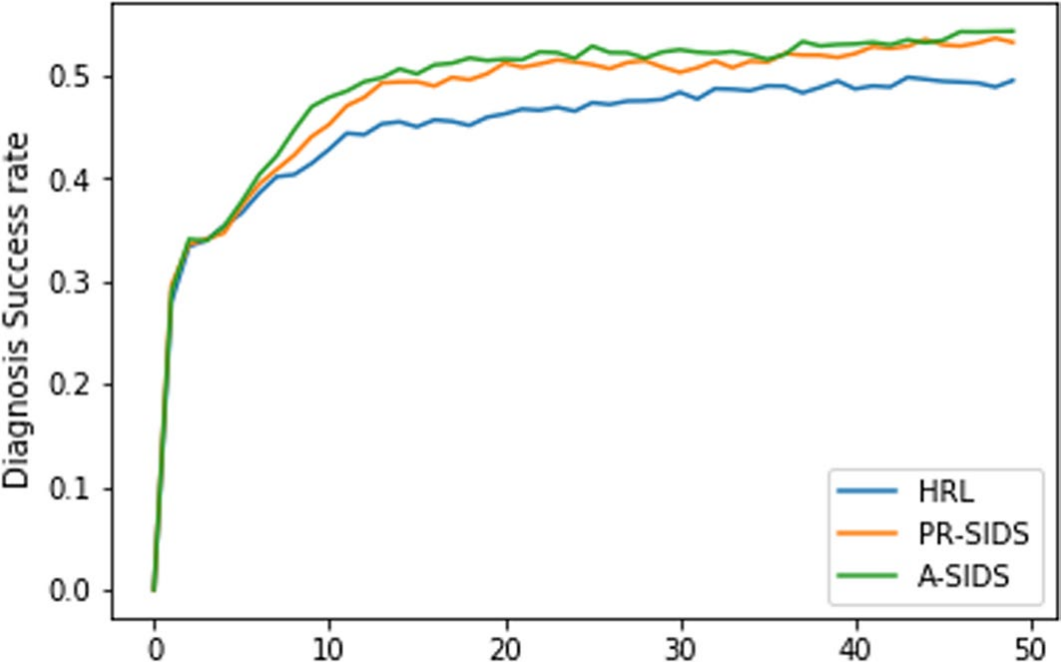
Diagnosis success rates of different agents over training episodes

Figure 5 shows diagnosis learning curves of different agents during training episodes. The performances of different models are reported in Table 2. Our model, A-SIDDA, outperforms the state-of-the-art methods in terms of all evaluation metrics by a significant margin. The performances of different agents with the DDQN algorithm are reported in Table 3. Our model still outperforms other baselines and state-of-the-art methods, but the diagnosis success rate and AMR are lesser and higher compared to DQN, primarily because of the reduced dialogue length.

**Table 2:** Performance of the proposed model and other baselines (average of five iterations) with Deep Q Network (DQN) algorithm as policy learning method

Model	Diagnosis success rate	Dialogue length	AMR (%)	AMR2 (%)	Disease classifier accuracy (DC) (%)
SVM-ex	0.3210	/	/	/	32.10
REFUEL Peng et al. [15]	0.3470	4.56	/	16.10	/
KR-DS Xu et al. [31]	0.3570	6.24	/	38.80	/
GAMP Xia et al. [14]	0.2670	1.36	/	7.70	/
Flat policy	0.3420	5.34	2.41	1.26	/
HRL Liao et al. [12]	0.5040	12.95	10.49	29.41	49.80
PR-SIDDA with only AM	0.5226	12.44	9.38	36.85	52.86
PR-SIDDA with only RM	0.5182	9.14	13.90	37.38	52.14
PR-SIDDA	0.5162	9.94	13.52	40.84	51.42
A-SIDDA with only AM	0.5260	11.69	9.75	33.70	53.53
A-SIDDA with only RM	0.5378	11.34	12.34	42.66	54.18
A-SIDDA	0.5576	11.79	12.68	43.82	56.16

AM and RM refer to the association module and recommendation module, respectively

**Table 3:** Performances of the proposed model and other baselines (average of five iterations) with Double Deep Q Network (DDQN) algorithm as policy learning method

Model	Diagnosis success rate	Dialogue length	AMR (%)	AMR2 (%)	Disease classifier accuracy (DC) (%)
Flat policy [10]	0.3370	5.43	2.82	1.32	/
HRL (Liao et al., 2020) [12]	0.4784	8.57	13.80	29.24	48.40
PR-SIDDA with only AM	0.4906	8.92	11.34	29.21	49.08
PR-SIDDA with only RM	0.4984	8.58	14.96	35.76	49.01
PR-SIDDA	0.4820	7.74	15.32	33.10	49.22
A-SIDDA with only AM	0.4737	8.73	11.40	27.50	47.31
A-SIDDA with only RM	0.4996	8.27	14.78	33.44	49.04
A-SIDDA	0.5201	8.34	16.04	40.54	52.52

AM and RM refer to the association module and recommendation module, respectively

**Table 4:** The left and right tables show the performances of the HRL agent and the proposed agent (A-SIDDA), respectively

Self-report	Self-report
Patient: Hi, I am having *difficulty in breathing*. What could be its reason ?	Patient: Hi, I am having *difficulty in breathing*. What could be its reason ?
*Conversation*	*Conversation*
*Agent*: Ok, are you feeling shortness of breadth ?	*Agent*: Ok, are you feeling shortness of breadth ?
*Patient*: Yes, I am feeling shortness of breadth.	*Patient*: Yes, I am feeling so.
*Agent*: Do you have skin rash ?	*Agent*: Have you vomited recently ?
*Patient*: No, skin rash.	*Patient*: Yes, I vomited a few times.
*Agent*: Have you vomited recently ?	*Agent*: Have you felt nausea ?
*Patient*: Yes, I vomited a few times	*Patient*: No, I have not felt so.
*Agent*: Have you observed abnormal appearing skin ?	*Agent*: Do you have cough ?
*Patient*: No, I am not suffering from skin issue.	*Patient*: No, I do not have cough.
*Agent*: You are most likely to have *Fluid overload*	*Agent*: You are most likely to have *Fluid overload*
*Diagnosis success : True, Dialogue length : 4, Match rate : 0.5,*	*Diagnosis success : True, Dialogue length : 4, Match rate : 0.5,*
*DC : 1, IReS-1 : 0.3337, IReS-2 : 0.9304*	*DC : 1, IReS-1 : 0.5271, IReS-2 : 1.121*

The significance of the agents’ investigated symptoms differs substantially, yet they perform identically in all current evaluation metrics (success rate, dialogue length, AMR, and DC). The proposed evaluation metric, IReS, successfully captures the relevance aspect and rates the symptom investigations accordingly

The underline signifies the medical entity which is being inspected

**Table 5:** Performances of different agents (average of five iterations) in terms of relevance of symptom investigation (IReS-1, IReS-2) and disease coverage

Model	IReS-1	IReS-2	Top 3 disease coverage	Top 5 disease coverage
HRL Liao et al. [12]	0.3058	0.4975	77.30	87.70
PR-SIDDA with only AM	0.2520	0.3565	81.02	91.90
PR-SIDDA with only RM	**0.4385**	0.7635	80.64	90.44
A- SIDS with only AM	0.2611	0.2987	81.40	90.96
A- SIDS with only RM	0.2938	0.4083	**82.82**	92.50
**A-SIDDA**	0.4123	**0.8458**	82.26	**92.74**

The bold figure shows superiority

We also evaluated these models in terms of symptom relevance (IReS-1, IReS-2) and condition coverage. The obtained results (Table 5) firmly establish the proposed association-based A-SIDDA model’s efficacy for conducting appropriate and relevant investigations, which are the key improvements over the state-of-the-art method. In Table 4, we also present a case study in which both agents perform identically in all existing evaluation metrics (Success, turn, AMR, and DC). However, the relevance of symptoms inspected by the SIDDA agent is substantially more relevant than the HRL agent, which had enquired some relatively irrelevant symptoms related to skin despite the patient informed breathing difficulty. Our proposed evaluation metric, IReS scores, captures this subjective concern and accordingly rates the symptom investigations.

**Fig. 6:**
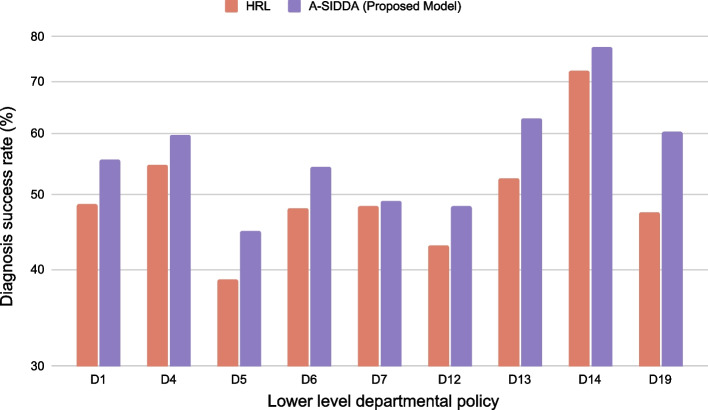
Diagnosis success rate of different departmental polices of HRL (brown) and proposed A-SIDDA model (purple)

Table 6 shows the performance of the proposed model across different disease departments. The performance comparison of these lower-level policies of the proposed model and the state-of-the-art model (HRL) has been illustrated in Fig. 6. The A-SIDDA model outperforms across all lower-level policies in terms of diagnosis accuracy by a significant margin.

**Table 6:** Performance of the proposed model across different disease departments

Department	Diagnosis success rate	Dialogue length	AMR (%)	AMR2 (%)
1	0.554	11.15	15.98	41.84
4	0.596	12.57	4.80	29.45
5	0.448	10.21	15.24	45.59
6	0.542	12.12	13.27	42.83
7	0.491	12.36	15.60	53.96
12	0.483	10.74	10.82	45.15
13	0.627	12.37	8.40	31.88
14	0.775	8.80	5.80	21.44
19	0.603	14.08	24.16	57.20

## Human evaluation

**Fig. 7:**
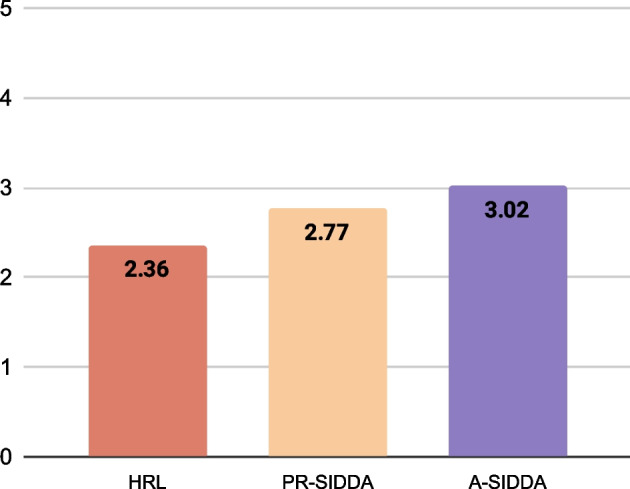
Human scores obtained by different diagnosis dialogue agents

To rule out the possibility of under informative assessment done by automatic metrics, we conducted the human evaluation of 100 randomly selected test samples. In this assessment, medical domain experts, including three researchers, out of which two are clinicians, have been employed to rate each diagnosis from 0 to 5 based on *investigation relevance, coherence, success, diagnosis time, and relevance of predicted disease*. The obtained average scores are reported in Fig. 7.

The proposed agent’s symptom investigation relevance is tremendously better than the HRL agent. The HRL inspects the most frequently occurring symptoms (skin rash, fever, cold) more often without considering the context. The proposed agent’s efficacy reduces in case of diseases with a huge number of symptoms as the association-based recommendation leads to a wider horizon.

*Key findings* The key findings and observations from the experiment are enumerated as follows: i. The proposed A-SIDDA outperforms existing diagnosis assistants by a significant margin in both policy optimization algorithms (DQN and Double DQN) and achieves state-of-the-art performance. ii. The A-SIDDA outperforms the PR-SIDDA because the PR-SIDDA considers only patient self-reports while recommending a symptom for inspection in each dialogue turn. As a result, it does not capture the ongoing dialogue context, whereas the A-SIDDA uses the entire discourse, including patient self-report, to make such a recommendation. iii. We also found that our proposed assistant performs superior in terms of disease coverage (Top 3/5 disease converge) and achieves an acceptable disease identification accuracy. iv. The PR-SIDDA with only RM performs best in terms of IReS-1 primarily due to the fact that IReS-1 measures the relevance of inspected symptoms with patient self-report only (Table 5). Here, the assistant that also incorporates the recommendation module (RM) always recommends a symptom based on only patient self-report, and thus it becomes less efficient for symptom investigation. v. We found that the distribution of performances of our agent and the existing state-of-the-art model (HRL) across departmental policies is similar. Our A-SIDDA outperformed the HRL agent in every department by a significant margin. vi. We also observe that the proposed diagnosis method not only improves diagnosis efficacy but also greatly enhances end-users satisfaction because of context-aligned relevant and minimal symptom investigation (Table 2 and Fig. 7).

## Case study and analysis

**Fig. 8:**
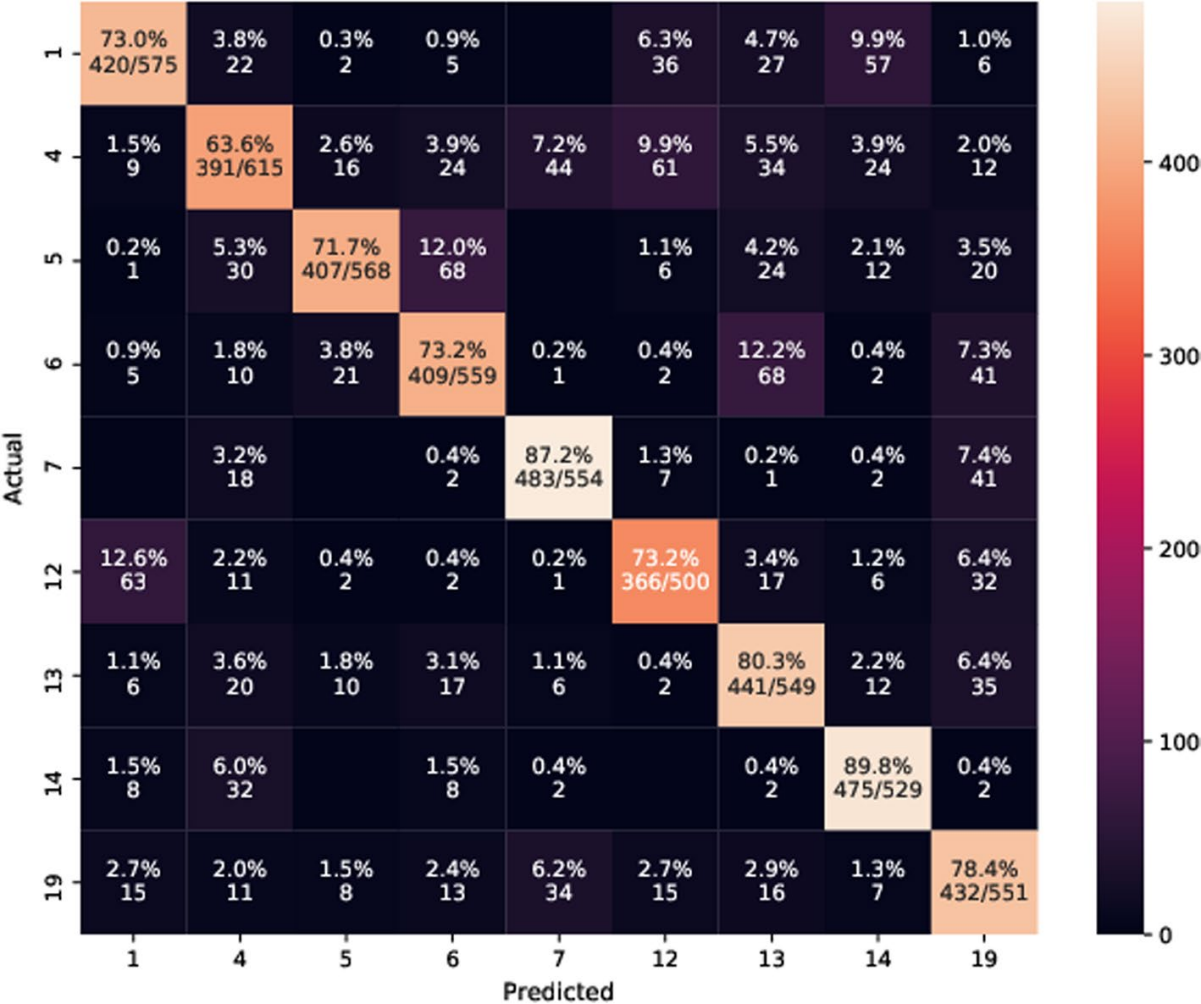
Confusion matrix for failed diagnoses - The diagonal elements show the percentage of times that the agent diagnoses an incorrect disease despite predicting the right disease group, i.e., the agent fails to distinguish among diseases of its corresponding disease group primarily because of many common symptoms

We have analyzed the performance of our proposed agents and the HRL agent for some common test cases (Table 7). Two such performance comparisons are reported in Table 8 and Table 9. The HRL agent inspects some completely irrelevant and frequently occurring symptoms (skin rash, skin lesion, eye pain) and informs the incorrect disease. The proposed model conducts a more relevant and appropriate symptom investigation guided and controlled by the ARM module and diagnoses patients correctly with fewer turns. The confusion matrix for failed diagnoses across disease groups is reported in Fig. 8. It shows that the diagnosis agent diagnoses an incorrect disease despite predicting the right disease group. It fails to distinguish among diseases of its corresponding disease group primarily because of many common symptoms.

**Table 7:** Testing samples - Group id indicates disease group (department) and self-report contains patient’s chief complaints /symptoms reported explicitly by the patient

Case id	Disease	Group id	Self report	Implicit symptoms
13947	Carpal tunnel syndrome	6	Arm pain: True	Loss of sensation: True, Hand or finger pain: True, Wrist pain: True, Neck pain: True
22285	Gas gangrene	1	Wrist pain: True	Sharp abdominal pain: True, Pain during pregnancy: True, Facial pain: True, Fatigue: True

**Table 8:** Performance on test case (Table 7, case id 13947) - The left table shows the performance of the HRL agent, which informs an incorrect disease after an investigation of 8 turns, and the right table illustrates the performance of the proposed agent (A-SIDDA), which diagnoses the patient correctly through a more relevant symptom investigation of only 7 turns

Self-report	Self-report
Patient: Hi, I am experiencing *arm pain* since few days. What could be its reason ?	Patient: Hi, I am experiencing *arm pain* since few days. What could be its reason ?
*Conversation*	*Conversation*
*Agent*: Do you also feel neck pain ?	*Agent*: Do you feel ache all over?
*Patient*: Yes, I am feeling neck pain.	*Patient*: No, I do not feel ache.
*Agent*: Are you experiencing any skin itching ?	*Agent*: Are you experiencing neck pain?
*Patient*: No, I do not have skin itching issue.	*Patient*: Yes, I do.
*Agent*: Have you observed skin lesion ?	*Agent*: Do you have headache?
*Patient*: No.	*Patient*: No.
*Agent*: Do you have skin rash problem ?	*Agent*: Are you feeling back pain?
*Patient*: No, I do not have this issue.	*Patient*: I do not have this problem.
*Agent*: Do you have eye pain ?	*Agent*: Have you felt loss of sensation?
*Patient*: No.	*Patient*: Yes, I am feeling loss of sensation.
*Agent*: Have you felt anxiety and nervousness ?	*Agent*: Have you experienced paresthesia?
*Patient*: I am not feeling anxious.	*Patient*: I have not felt paresthesia.
*Agent*: Do you have facial pain ?	*Agent*: Are you feeling leg pain?
*Patient*: No.	*Patient*: I do not have leg pain.
*Agent*: Are you feeling leg pain ?	*Agent* : You are most likely to have *carpal tunnel syndrome*
*Patient*: No, I am not feeling leg pain.	
*Agent* : You are most likely to have *Adhesive capsulitis of the shoulder*.	

The underline signifies the medical entity which is being inspected

**Table 9:** Performance on test case 2 (Table 7, case id 22285) The left table shows the performance of the HRL agent, which informs an incorrect disease (Chancroid) after an investigation of 8 turns, and the right table illustrates the performance of the proposed agent (A-SIDS), which diagnoses the patient correctly through a more relevant symptom investigation of only 6 turns

Self-report	Self-report
Patient: Hi, I am feeling *wrist pain*. Can you please assist me ?	Patient: Hi, I am feeling *wrist pain*. Can you please assist me ?
*Conversation*	*Conversation*
*Agent*: Are you also feeling hand or finger pain ?	*Agent*: Sure, do you feel pregnancy pain ?
*Patient*: No, I am not feeling Hand or finger pain.	*Patient*: Yes, I feel pregnancy pain.
*Agent*: Do you feel lower body pain ?	*Agent*: Do you have facial pain ?
*Patient*: I do not have lower body pain.	*Patient*: Yes, I do have facial pain.
*Agent*: Ok, are experiencing lower back pain ?	*Agent*: Ok, are you feeling any eye pain ?
*Patient*: No, I do not think so.	*Patient*: No.
*Agent*: Do you feel pregnancy pain ?	*Agent*: Have you noticed excessive urination at night ?
*Patient*: Yes, I am experiencing pregnancy pain frequently.	*Patient*: I do not feel so.
*Agent*: Ok, are you feeling excessive urination at night ?	*Agent*: Do you have shoulder cramps or spasms ?
*Patient*: I do not think so.	*Patient*: No.
*Agent*: Are you experiencing facial pain ?	*Agent*: Do you feel sharp abdominal pain ?
*Patient*: Yes, I am feeling facial pain.	*Patient* Yes, I am suffering from sharp abdominal pain.
*Agent*: Have you noticed *skin lesion* ?	*Agent* : You are most likely to have *Gas gangrene* based on these symptoms.
*Patient*: No.	
*Agent*: Are you observing *abnormal appearing skin* ?	
*Patient*: No, I do not have any skin issue.	
*Agent*: You are most likely to have *Chancroid* based on these symptoms.	

The underline signifies the medical entity which is being inspected

*Strengths and limitations* The key strengths of the proposed association guided symptom investigation and diagnosis assistant (SIDA) are as follows: **i.** The proposed assistant diagnoses patients more accurately and in less time than any existing disease diagnosis assistants (Table 2). **ii.** The proposed assistant conducts symptom-association guided symptom inspection, i.e., each symptom inspection is inspired by ongoing dialogue context (previously confirmed symptoms). As a result, the conducted symptom inspection used to be quite pertinent to patient discourse and their primary complaint, making it outperform current diagnosis assistants in both quantitative and qualitative evaluations. **iii.** We first propose an evaluation metric, IReS, for measuring the relevance of symptom investigation conducted by a conversational assistant. Our proposed SIDA outperforms existing state-of-the-art models in all evaluation metrics, including IRes (Table 5). This metric can be utilized to evaluate a diagnostic agent’s effectiveness based on the relevance of the symptoms examined during symptom investigations.

The proposed methodology also has some weaknesses, which are as follows: **i.** Our key novelty lies in the central module of the dialogue system, i.e., dialogue management and dialogue policy learning [45]. The proposed framework utilizes a template-based response and diagnosis report generation. So, it may reduce an end-user’s interest due to monotonous responses. Hence, a neural context-aware generation method could be incorporated to generate context-aware engaging responses. **ii.** In some cases, the proposed assistant inspects significantly more symptoms than the existing diagnosis assistant (HRL) in order to arrive at a conclusive diagnosis. The primary reason for the behavior is the association-based critic, which encourages the assistant to continue inspecting more symptoms if it observes a patient’s symptom that co-occurs with a large number of symptoms. **iii.** In the proposed diagnosis setting, the agent assumes that end-users are familiar with medical entities such as symptom names. However, a large population is unacquainted with many symptoms, such as mouth ulcer and skin growth. Furthermore, some signs/symptoms are hard to express through text. As a result, a multimodal disease diagnosis that allows end users to express their symptoms through both text and images can be more effective and user-satisfying.

## Conclusion

With the constantly expanding human population, the public healthcare system and health professionals are under strain. Thus, both research and industry communities are experiencing an ever-growing demand for artificial intelligence based tools and techniques for automatizing medical operations. Motivated by the importance of symptom association in the diagnosis process, we investigated the role of symptom association and built an association guided symptom investigation and disease diagnosis assistant (A-SIDDA). The proposed diagnosis model consists of a two-layered hierarchical policy structure, an association & recommendation module (ARM), and a disease classifier. The higher level policy decides medical department, and the lower level policy conducts department-specific symptom investigation. The ARM module reinforces the assistant to conduct context-aligned association guided symptom inspection through symptom recommendation and an additional critic. The disease classifier identifies a disease based on the patient’s self-report and the additional extracted symptoms. Furthermore, we introduced a novel evaluation metric called Investigation relevance score (IReS) to evaluate the relevance of symptom investigation, which estimates end users’ satisfaction with the system. The proposed assistant suppresses several baselines and state-of-the-art methods across multiple policy optimization algorithms by a significant margin and achieves state-of-the-art performance. We also conducted a human evaluation of the behavior of different diagnosis assistants, and we observed that the proposed assistant enhances user satisfaction significantly because of context-aligned symptom investigation. The obtained improvements (both quantitative and qualitative) firmly evidence the crucial role of symptom association and its usefulness in the diagnosis process. In future, we would like to develop a multitasking diagnosis framework that inspects symptoms and diagnoses disease using a unified network. We would also like to investigate the importance of signs/symptoms communicated through visuals in diagnosis process.

## Data Availability

The data and the code will be made publicly available through the Github repository - https://github.com/NLP-RL/A-SIDDS.

## Footnotes

1 https://www.mayoclinic.org/.

2 https://www.babylonhealth.com/en-gb.

3 Division has been done as per the International Classification of Disease called ICD-10-CM https://www.cdc.gov/nchs/icd/

4 The data and code are available at https://github.com/NLP-RL/A-SIDDS

5 The model was trained for 5000 episodes on an RTX 2080 Ti GPU, which took around 22 hours
